# Omsk Hemorrhagic Fever Virus: A Comprehensive Review from Epidemiology to Diagnosis and Treatment

**DOI:** 10.3390/microorganisms13020426

**Published:** 2025-02-15

**Authors:** Erica Diani, Riccardo Cecchetto, Emil Tonon, Marco Mantoan, Virginia Lotti, Anna Lagni, Asia Palmisano, Pier Paolo Piccaluga, Davide Gibellini

**Affiliations:** 1 Department of Diagnostics and Public Health, Microbiology Section, University of Verona, 37134 Verona, Italy; riccardo.cecchetto@univr.it (R.C.); emil.tonon@univr.it (E.T.); virginia.lotti@univr.it (V.L.); anna.lagni@univr.it (A.L.); asia.palmisano@studenti.univr.it (A.P.); davide.gibellini@univr.it (D.G.); 2 UOC Microbiology Unit, AOUI Verona, 37134 Verona, Italy; 3 Department of Diagnostics and Public Health, Section of Hygiene and Preventive, Environmental and Occupational Medicine, University of Verona, 37134 Verona, Italy; marco.mantoan@univr.it; 4 Biobank of Research, IRCCS Azienda Ospedaliera-Universitaria di Bologna Policlinico di S. Orsola, 40138 Bologna, Italy; pierpaolo.piccaluga@unibo.it; 5 Department of Medical and Surgical Sciences, Bologna University School of Medicine, 40138 Bologna, Italy

**Keywords:** Omsk hemorrhagic fever virus, flaviviridae, hemorrhagic fever, TBEV, tick-borne encephalitis complex

## Abstract

Omsk hemorrhagic fever virus (OHFV) is the etiological agent of a poorly studied acute viral disease, causing several epidemic waves observed in the western Siberia regions of Omsk, Kurgan, Novosibirsk, and Tyumen. OHFV is a flavivirus and shares structural and morphological features with tick-borne encephalitis (TBE) complex viruses. The disease’s symptoms show high variability, from flu-like symptoms, hyperesthesia, and petechial rush in the upper body to high fever and hemorrhagic manifestations, with a fatality rate of about 1%. The real number of OHFV-infected people is still unknown due to the difficulties in diagnosis and the presence of asymptomatic patients that lead to an underestimation of the total cases. Little is known about the viral infection dynamics at the molecular and cellular levels, the viral involvement in immune escape, cellular pathways alteration, or metabolic influence. It is noteworthy that no clinical trials have currently been performed for effective and specific drug treatments. In this review, we will give an overview of OHFV interactions with humans and animals, diagnostic tools, and drug treatments. We aim to highlight the importance of a frequently undiagnosed or misdiagnosed viral infection that might also even cause severe clinical manifestations such as meningitis and hemorrhage, in order to point out the need to develop new research studies, new diagnostic tools, and new treatments for OHFV.

## 1. History

The history of OHFV began in 1944, when several cases of a new type of hemorrhagic fever that differed from other manifestations related to different viruses, including Crimean hemorrhagic fever, were detected in the Russian Omsk oblast [1]. After the beginning of a third large epidemic of this new disease in the same endemic area in 1947, a scientific expedition under the leadership of Professor Chumakov, from the Academy of Medical Sciences of the USSR, including military medical officers, entomologists and microbiologists, was able to define the following: (i) the viral etiology, (ii) the difference between the causative virus and other viruses known to cause hemorrhagic fever events, (iii) the mechanism of viral transmission, and (iv) the viral pathogenesis [2]. In particular, the first strain of OHFV was isolated, and viral transmission was associated with arthropod vectors that acted as intermediate hosts between infected animals, mainly muskrats, and humans. Starting from this expedition in 1947, other epidemic peaks were described in the Omsk oblast and in the surrounding areas. Subsequently, viral spread was detected in other areas (in the western Siberia regions of Novosibirsk, Kurgan, and Tyumen and, more recently, in Kazakhstan [3,4,5]) due to climate change and animal importations [6].

This review will give an overview of a little-known virus that may cause severe symptoms in humans. This is important as the common clinical features between OHFV and TBEV often lead to misdiagnosis. Considering climate change and the consequent changes in the seasonal and geographical diffusion of animal and arthropod vectors of OHFV, and its high similarity with tick-born encephalitis virus complex (TBEV), we can expect a higher diffusion in the coming years. With this review, we aim to increase awareness of OHFV and describe the state of the art of its prevention and treatment.

## 2. Viral Genome and Structure

**Figure 1:**
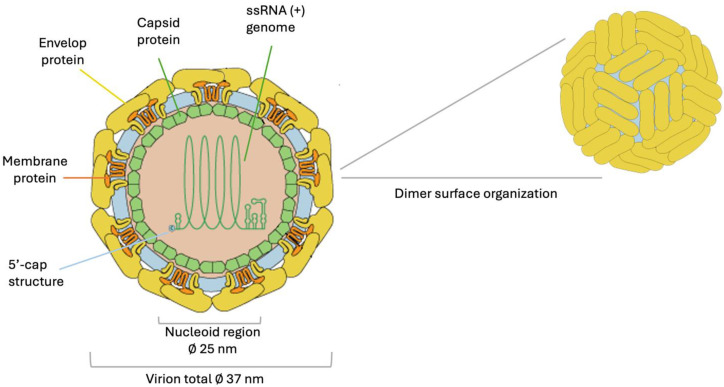
The structure of the flaviviridae virion with specific features of OHFV. Due to the lack of a schematic representation of the OHFV external structure, we added specific OHFV features to a common Flaviviridae virion scheme. Image modified by ViralZone, SIB Swiss Institute of Bioinformatics (https://viralzone.expasy.org/ accessed on 6 February 2025).

OHFV is a flavivirus belonging to the *Flaviviridae* family, closely related to the TBE virus complex [7]. This viral group shares high genomic and morphological similarity. At a morphological level, the structure of OHFV was described in an electron microscopy study published in 1966 as an enveloped virus with a spherical or polygonal shape, with a diameter of about 37 nm characterized by a hyperdense nucleoid about 25 nm in diameter [8]. To our knowledge, the OHFV external structure has not been described, even though it is conceivable that the OHFV virion is similar to other Flaviviridae (Figure 1).

**Figure 2:**
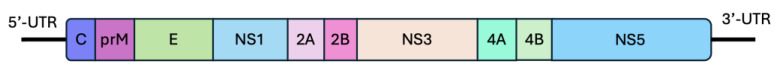
Schematic representation of OHFV viral genome organization.

The genome is represented by a positive-sense single-stranded RNA genome (Figure 2) of 10,787 nucleotides that contains only one open reading frame (ORF) of 10,242 nucleotides flanked by two untranslated regions (UTRs) at both the 5′ and 3′ termini [9]. The 5′-UTR is shorter than the 3′-UTR and is characterized by a particular secondary structure with two stem-loops spanning a region of 123 nucleotides. It is noteworthy that the OHFV genome shows a 5′-cap structure, which is absent from other tick-borne flaviviruses [9].

OHFV, unlike other TBEV Western subtype strains, lacks a long terminal poly(A) tract. The variable region instead shows high heterogenicity compared to other TBEV genomes, indicating a marginal role in viral functions [10]. In particular, in the major stem-loop structure located in the initial region of 5′-UTR, the OHFV genome shows approximately 30 nucleotides that differ from other TBE complex viruses. The remaining 5′-UTR is highly conserved. Lin et al. suggest a role of this region in vector- or tissue-specific OHF virus replication [9]. On the other hand, the 3′-UTR, consisting of 413 nucleotides, is more conserved in the TBEV group and contains a variable region, in length and sequence, and a core highly conserved region [9]. Also, in the OHFV genome, the 3′-UTR core region is highly homologous to other TBEVs and should play a key role in infectivity, as observed in TBEVs [10,11].

The ORF encodes for a polyprotein that is cleaved by host and viral proteases during translation. All cleavage sites are highly conserved in the OHFV viral genome. Polyprotein organization shows the three classical structural proteins: the capsid (C), the preM (prM), and the envelope (E), and seven non-structural proteins [12].

Through the alignment of the entire viral genome with other TBEVs, Bondaryuk and colleagues [13] determined that OHFV had a very high substitution rate, compared with other RNA viruses, with a value between 10^−5^ and 10^−4^ substitution per site per year (s/s/y) [14], indicating its considerable mutation rate related to RNA polymerase RNA-dependent functional characteristics. This is a classical feature of Flavivirus that influences the development of specific antiviral molecules and vaccines. In the same study, Bondaryuk and coworkers aligned 17 ORF regions obtained from 43 complete genome sequences of OHFV and suggested classifying the viral genomes into three different genotypes as previously suggested [15]: twelve isolates were assigned to genotype 1, a single isolate to genotype 2, and four isolates to genotype 3. They also reported genotype 1 to be older than genotype 3, with the latter diverging from genotype 2 approximately 461 years before their analysis. All other isolates remained unclassified [13]. The same study also compared the data obtained from the alignment of viral ORF sequences with those achieved from the alignment of 75 different E viral gene sequences. This comparison is interesting since it shows that the mutation rate evaluated using an analyzing E viral gene sequences can obtain reveals a relatively confident value for viral evolution, as with the ORF data [13].

**Figure 3:**
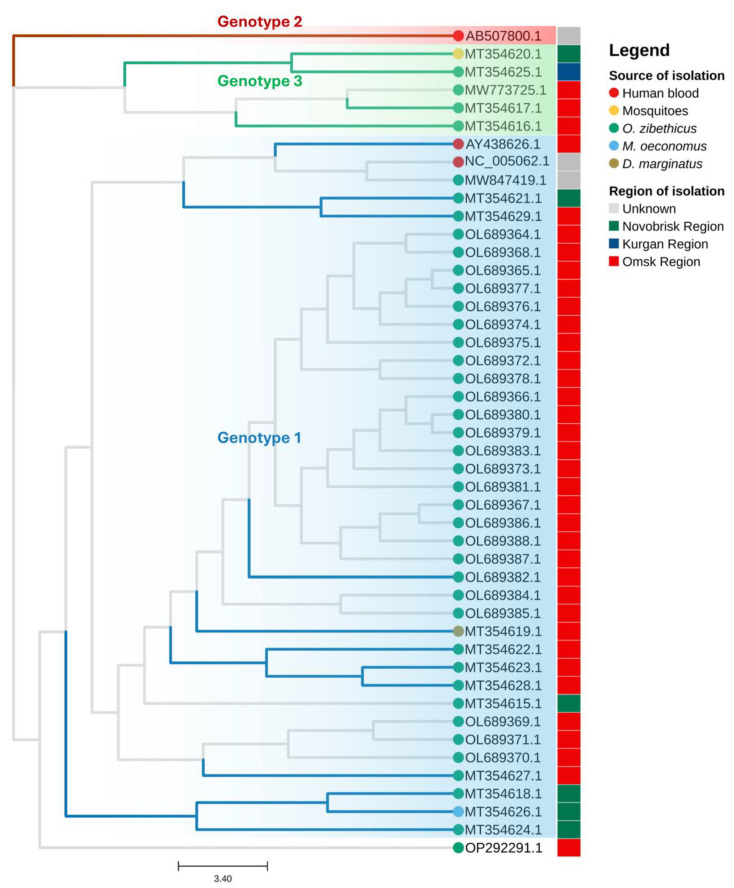
OHFV ML phylogenetic tree obtained with MAFFT v7, IQ–TREE software version 2.4.0 and visualized with Tree Viewer v2.2.0 of 46 viral complete sequences of different strains of OHFV. Branches in blue are classified as genotype 1, branch in red as genotype 2, branches in green as genotype 3; branches unclassified in the literature are shown in gray [13]. In the same color shading are the strains that can be classified to that specific genotype. Dots of different colors represent the source of isolation: red for strains isolated from human blood, yellow for strains isolated from mosquitoes, green for strains isolated from *O. zibethicus*, light blue for strains isolated from *M. oeconomus*, and brown for strains isolated from *D. marginatus*. Boxes of different colors represent the region of isolation: in green, the strain isolated in the Novosibirsk region; in blue, the strain isolated in the Kurgan region; in red, the strains isolated in the Omsk region; and in gray, the strains of the unknown region of isolation.

Up to January 2025, 49 complete genome sequences for 46 different strain types had been uploaded onto the NCBI database. Strains were isolated in different Russian regions between 1947 and 2007 in muskrats (*Ondatra zibethicus*), rodents (*Microtus oeconomus*), and arthropods (mosquitoes and *Dermacentor reticulatus*). In 2003, the first complete genome of the OHFV Guriev strain was sequenced. We reconstructed a maximum likelihood (ML) phylogenic tree (see Figure 3) for all 46 unique strains present in the NCBI database.

Bondaryuk and colleagues conducted a phylogenetic analysis on 17 OHFV complete sequences, classifying them into three different genotypes: 

We reconstructed a phylogenetic tree using all 46 known strains of OHFV complete genome sequences uploaded to NCBI and obtained three different clusters, corresponding to the three genotypes. However, as unclassified strains cluster within the same branch as genotyped strains, they can be assigned to the same genotype based on their phylogenetic placement. According to our tree (Figure 3), an additional 28 strains can be classified as genotype 1, and 1 as genotype 3.

In addition, the genotype 2 strain was only isolated from human blood; more isolates are needed to establish whether this genotype has an enhanced human tropism. The year and location of isolation are reported in Table 1, as well as the strain and genotype, the organism of isolation, and the sequencing date.

No isolates have been sequenced directly from human samples. The newest samples were isolated in 2007. More recent specimens could provide additional information on genotype spreading and genome evolution.

## 3. Geographical Distribution

**Figure 4:**
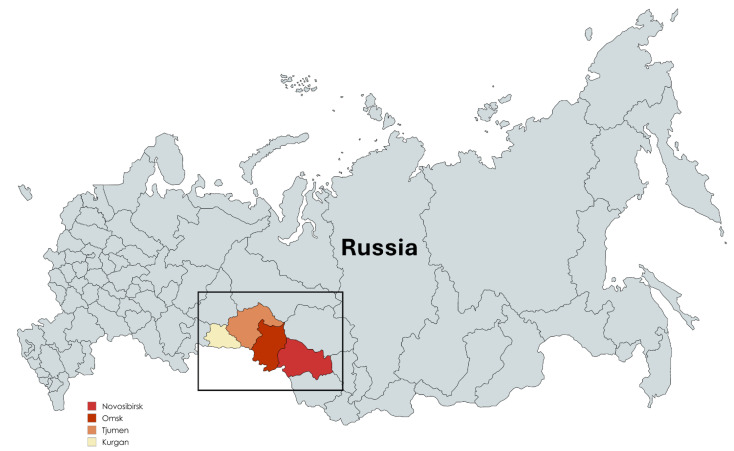
Geographical distribution of Omsk hemorrhagic fever. Endemic administrative regions of Russia. This graph was modified from the CDC guideline website [16] and created with MapChart (http://www.MapChart.net, accessed on 6 February 2025).

The natural reservoir of OHFV is limited to the forest steppe and lake regions of Omsk, Kurgan, Novosibirsk, and Tjumen (as shown in Figure 4). These regions show common features, which provide the natural habitat for many *Ixodidae* ticks and muskrats, which represent the most important targets in OHFV infection. Unfortunately, the exact distribution of OHFV in other regions (i.e., Tomsk, Altai, and Kemerovo) is still not fully determined.

Different geographic and seasonal distributions of ticks are closely linked to viral transmission, their reservoirs, and the viral mutational rate. Geographically, the principal tick species in the Northern region is represented by *Dermacentor marginatus*. In the Southern region, *Dermacentor pictus* is predominant, while *Dermacentor reticulatus* serves as the primary viral reservoir in the forest steppe region of Siberia.

In addition to these ticks, two different families of mites, a mesostigmatic family and a water family called *Gamasidae* and *Hydracarinae*, respectively, could play a role in OHFV transmission and virus maintenance [17]. The life cycles of ticks are linked with the seasonal transmission of OHFV: adults are mainly active during March and April, showing a decrease during the summer and a second activity peak between September and October [18,19,20].

Because *D. reticulatus* has evolved some key characteristics, including a high reproductive rate, the rapid completion of its life cycle, underwater survival for months, and a wide host range [21], the transmission of OHFV outside Russia has been made possible. Thus, Wagner and colleagues recently reported the incidence of OHFV outside Russia, in the Republic of Kazakhstan [22]. It has been hypothesized that the virus may spread outside endemic areas due to several factors, particularly climate change, mammals and birds migration that may carry tick vectors [23].

The distribution of ticks during epidemic waves is influenced by the presence of rodents that host nymphs and larvae; a decrease in the number of rodents led to a significant decline in the arthropod vector population and a consequent decrease in viral transmission. The great epidemic of 1945–1947 was caused by a significant increase in the number of *Ondatra zibethicus*, a species of muskrat that was introduced in the Omsk region from Canada about 20 years before and reached its peak in the 1940s, with a correspondingly high number of cases of leptospirosis, tularemia, and hemorrhagic fever [24].

Notably, there is not a unique, commonly accepted hypothesis explaining the origin and the spreading of OHFV. The clues to be taken into account are the site of the first cases of OHFV infection and the initial widespread distribution of muskrats in Omsk oblast. The first hypothesis suggests that OHFV was imported as a new virus from the USA and Canada through the introduction of 4340 specimens of *Ondatra zibethicus* muskrats into the Siberian region. Another hypothesis suggests that OHFV, sharing some hemorrhagic characteristics, evolved from the Kyasanur Forest disease virus found in India. However, the most plausible current hypothesis, supported by Bondaryuk’s studies, suggests that OHFV is a new viral species that originated from TBEV [3,13].

## 4. Viral Transmission

OHFV is transmitted principally by an arthropod vector, usually a tick that has taken a blood meal from an infected host and then bites humans and animals [13]. Human-to-human transmission has not been reported, and it seems to be unlikely. Human transmission is also reported via direct contact with infected animals through the respiratory route or by infected milk ingestion [25].

Reservoir hosts for infection by ticks include muskrats and local voles (*genus Arvicola*), some of which undergo benign infection. The virus can infect other muskrats or humans via the blood, feces, and urine of infected muskrats in swamps or by leeches (*Glossiphoniidae*) moving into the water from dead muskrats. Although the presence of OHFV has also been reported in mosquitoes and water birds, they do not play an important role in the spread of the virus [26].

**Figure 5:**
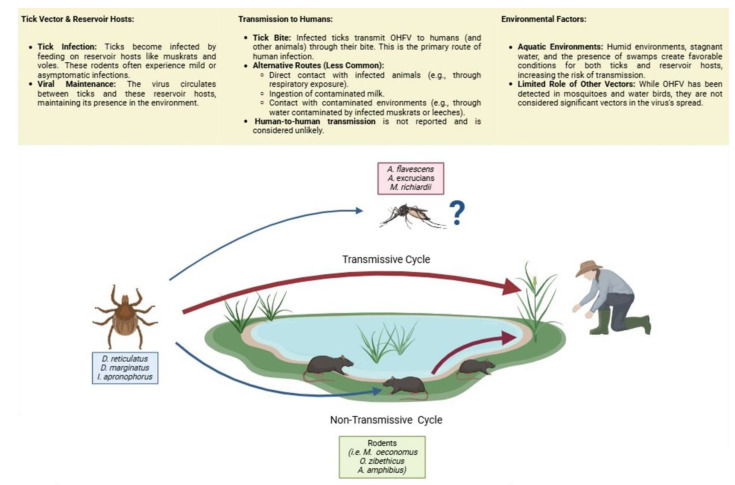
Transmission route of the ecological cycle of OHFV. In the graphic, the principal hosts and vectors involved in OHF infection are shown. OHFV is primarily found in rodents and is spread by the bite of an infected tick (non-transmissive cycle). Similarly, humans can contract the disease following an infected tick bite or by contact with biological material from an infected, sick, or deceased animal (transmissive cycle). Hunting and trapping are examples of recreational and occupational activities that may raise the risk of infection in humans. Human-to-human transmission has not been reported to date. Created with bioRender.com (http://www.biorender.com, accessed on 6 February 2025).

The main primary hosts of OHFV are rodents that are infected through a bite from an arthropod vector, such as *Dermacentor reticulatus*, *Dermacentor marginatus*, or *Ixodes persulcatus* species. The major rodent species involved are muskrats of the species *Ondatra zibethicus* (imported from Canada in the 1940s), narrow-skulled voles (*Stenocranius gregalis*), and water voles (*Arvicola amphibius*). Due to the preference of mosquitoes and ticks for humid environments and the presence of stagnant water, it must be considered that the risk of tick bites, and therefore of viral transmission, increases for people involved in activities in aquatic or humid environments. A scheme of the transmission cycle and principally species involved is represented in Figure 5.

Transmission between ticks could occur if two ticks, one infected and one uninfected, simultaneously bit the same vertebrate host in close proximity [27].

### Virion–Host Cell Interactions

OHFV interaction and membrane fusion with target host cells is mediated by Envelope protein. E protein is organized into three main domains: ED1, ED2, and ED3. In particular, the ED3 domain is highly immunogenic and contains specific epitopes capable of binding to antibodies [28]. Located in the N-terminal part of protein E, ED3 seems to play a key role in the virus’s ability to interact with host cellular receptors, and variation in this domain across different flaviviruses could impact the specificity in receptor binding [29]

It has been noticed that mosquito-transmitted flaviviruses often exhibit an RGD/E motif (arginine- glycine- aspartic acid/glutamic acid) on the surface of ED3. This motif, which is absent in tick-borne flavivirus, could facilitate interaction with the host integrin protein.

Specific residues have been identified as being crucial in the infection process. Residues 306, 310, 364, and 366 are located near the upper surface of ED3 and are directly exposed to the solvent. In tick-borne flaviviruses, residue 306 is frequently a methionine, whereas in mosquito-borne flaviviruses, it is typically a valine. Differences in residues 364 and 366 have also been observed, specifically in OHFV I364M and N366S mutations; these variations may alter the infection process by affecting receptor interactions or other critical steps in viral entry. Thus, it is possible that these small variations in ED3 residues can influence the virus’s ability to trigger specific responses in the hosts, potentially impacting its infectivity and pathogenicity [29].

According to similarity studies on the UniProt database, there are some strategies that OHFV could employ to evade immune response. Following the viral cycle timeline, the first strategy is the inefficient cleavage of prM-E heterodimers, the phase prior to E dimer formation that is essential in correct envelope assembly. The maintenance of prM-E heterodimers leads to the formation of many immature OHFV virions. NS1 viral protein is secreted as a subunit of a lipoprotein complex. It can exert a role in immune evasion through the contrast of the complement function, interactions with dendritic and macrophage cells, and the inhibition of signaling pathways activated by Toll-like receptor 3.

## 5. Epidemiology

OHFV is transmitted to small mammals by *Dermacentor* spp. ticks in the Omsk, Novosibirsk, Kurgan, and Tyumen regions in the forest steppe zones of western Siberia. However, the disease clearly emerged when muskrats were introduced to the area to start a fur industry [30,31]. The morbidity of OHFV is characterized by seasonal peaks, with the first cases (about 1% of annual cases) usually occurring in April and the incidence reaching its highest peak in May and June (73% of total annual cases), which correlates with *D. reticulatus* activity in the northern forest steppe regions. The second and lower peak of incidence occurs between August and September (about 21% of total annual cases), likely due to the activity of *D. marginatus* in the southern and western areas of Siberia [32]. The morbidity declines in July.

**Figure 6:**
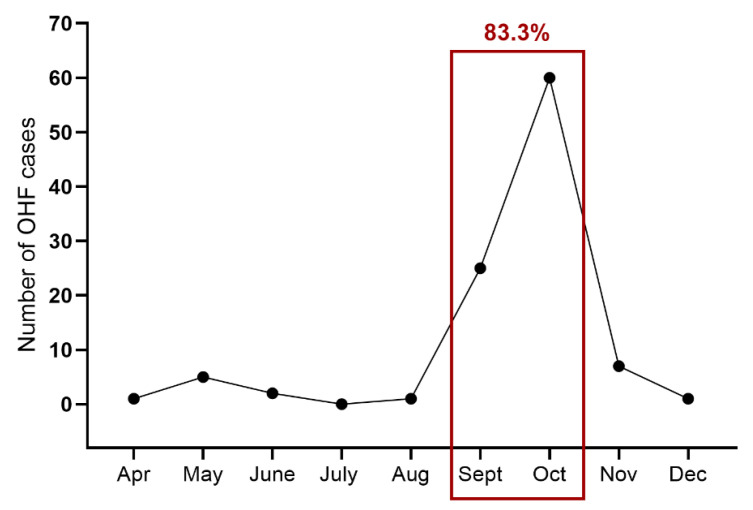
Cumulative seasonal variability in the morbidity of OHFV in the 1988–1992 outbreaks reported in the Novosibirsk region. Most of the cases (83.3%) were reported between September and October, corresponding to the muskrat hunting season (figure modified from Ruzek and coworkers [32]).

As confirmed by the 1988–92 OHFV outbreak data, 83.3% of all infections occurred between September and October (Figure 6) [33]. OHFV outbreaks are a common occurrence among individuals who have come into contact with muskrats, typically during the autumn or winter (from August to December), which coincides with the muskrat hunting season [34].

Between 1945 and 1958, OHFV outbreaks resulted in muskrat epizootics, and more than 1000 human cases were diagnosed, mostly among trappers, their relatives, and laboratory staff. The number of cases is likely to have been much higher than the number officially reported, since mild and asymptomatic cases were not always recorded [35,36]. The highest rate of incidence of Omsk hemorrhagic fever was in people aged 20–40 years, while a third of all cases were reported in children younger than 15 years.

Between 1960 and 1970, the incidence decreased remarkably, though the size and number of foci were thought to be declining. Indeed, in the past 30 years, OHFV has only been reported in the Novosibirsk region. In 1988, three cases were officially recorded in muskrat hunters. In 1989, 22 patients were diagnosed with OHFV, with the largest outbreaks reported in 1990 (29 cases) and 1991 (38 cases) [33]. Between 1988 and 1997, a total of 165 cases of Omsk hemorrhagic fever were reported. Of these cases, 10 were linked to tick bites, and 155 were discovered among muskrat hunters and poachers [35].

Nevertheless, the exact numbers of OHFV infections occurring every year remains unclear due to mild cases frequently being misdiagnosed or not even reported.

The spread of OHFV could be influenced by various socio-economic and ecological factors. These factors can affect the distribution and abundance of ticks, as well as human exposure to the virus. Ecological factors like temperature, humidity, and vegetation cover play a crucial role in determining the specific interactions between vectors and hosts in tick-borne diseases, ultimately affecting the epidemiological risks linked to ticks. These dynamics are made even more complex by climate change, which is likely to affect tick distribution and activity periods, expanding the geographic range of ticks and increasing the time window for tick-borne disease transmission [37]. Warmer temperatures and increased precipitation can prolong periods of tick activity by increasing vegetation growth, which provides more habitats for both ticks and their animal hosts. In addition, warmer winters can lead to fewer ticks being killed, allowing higher survival rates. Changes in seasonal patterns further exacerbate these effects, as longer warm seasons can prolong the duration of human exposure to ticks, thus increasing the risk of tick bites and disease transmission [38,39]. Ecological changes, such as logging and building expansion, have also led some species of rodents to occupy areas close to waterways and lakes, acting as a natural reservoir of this virus and favoring the spread of outbreaks [22].

## 6. Pathogenesis and Clinical Manifestation

The pathogenesis of OHFV infection is poorly understood, and the dynamics of immune response is not known. According to some studies, the virus shows both hematopoietic and vascular tropism. OHFV pathogenesis has been studied *in vivo* in non-human primates and laboratory mice. According to a study, BALB/c mice infected with OHFV experienced severe cerebellar abnormalities and mild meningoencephalitis. OHFV can overcome the blood–brain barrier and infiltrate the brain, causing a rise in IFN production in the central nervous system in newborn mice. Furthermore, edema and sensory abnormalities were detected in infected mice, with the onset of collapse and shock in some cases [32,40]. It was also demonstrated that OHFV infection in mice caused pneumonia, paralysis, and meningoencephalitis with a high viral load in the cerebellum, as well as other pathological signs such as splenomegaly and damage to the gastrointestinal tract, kidneys, and liver. Viral persistence in infected tissues and internal organs is a defining characteristic of pathogenesis in mice [8].

Laboratory findings reveal a severe neuronal infection in susceptible wild muskrats following an extraneural or intracerebral injection. Other wild vertebrates generally exhibit asymptomatic illnesses associated with viremia, including the Norway rat (*Rattus norvegicus*), water vole (*A. amphibius*), striped field mouse (*Apodemus agrarius*), and many others. Some infected animals showed reduced movement, weakness, and hyperpnea [32]. In contrast, infected macaques had no evident clinical or histological symptoms, and no virus was detected in their blood [41].

OHFV infection in humans can cause mild and flu-like symptoms, such as fever, cephalalgy, myalgia, and cough, but also moderate-to-severe hemorrhagic symptoms, pneumonia, nephrosis, and meningitis. The patients rarely experience chronic consequences, such as fatigue, hearing loss, hair loss, and behavioral and psychological difficulties connected with neurological disease. Prolonged resting is recommended in order to reduce the risk of long-term organ damage [33,42]. In fatal cases, death has occurred after hemorrhagic and septic complications.

Generally, signs and symptoms of Omsk hemorrhagic fever include hematological manifestations such as leucopenia, neutrophilia, monocytosis, and thrombocytopenia; dermatological manifestations such as hyperemia of the face and upper trunk, exanthema, and petechial rash; pulmonary manifestations with atypical pneumonia; renal manifestations with moderate albuminuria and intermittent hematuria; neurological signs such as nuchal rigidity and a decrease in sensorial stimuli perception (taste, hearing); and hemorrhagic manifestations, leading to nasal, pulmonary, gastrointestinal, and uterine hemorrhages [32]. In a subsequent outbreak (1988–1989), more than 80% of patients displayed a mild clinical form with no hemorrhagic disease and a death rate of about 1% [43,44]. The usual course involves 1–2 weeks of symptomatic sickness, in which 50–70% of patients recover without complications. On the contrary, in 30–50% of patients, a secondary phase occurs with persistent high fever, the recurrence of major symptoms, and the development of encephalic symptoms, persisting for 5–14 days. In these cases, internal organs are frequently damaged, particularly the lungs and kidneys, with bronchitis, pneumonia, or both in one third of cases. Symptoms of diffuse encephalitis often fade, along with other second-phase symptoms [22,44].

## 7. A Model for OHFV Studies

In 2005, Holbrook and colleagues [40] established an *in vivo* animal model to study and compare the effects of two different viral infections caused by two tick-born encephalitis viruses: OHFV and Powassan Virus (POWV). They injected a lethal dose of OHFV and POWV intraperitoneally into BALB/c female mice of 3–5 weeks old, and they observed that these infections produced similar symptoms to those in humans and could be well distinguished and characterized, proving that the BALB/c mice could be considered a good model for studying OHFV infection in humans. The Holbrook study could be improved with the comparison of mouse infection with other OHFV strains and at different viral concentrations, but it represents a good starting point to better define the viral mechanisms and pathogenesis.

In a more recent study [45], the same group demonstrated that C57BL/6 8–10-week old female mice could also be efficiently infected with OHFV. In this second murine model, however, the symptoms appeared milder, suggesting that the severity of the pathology varies depending on the host response. The Tigabu study highlighted that OHFV does not cause frank encephalitis in newborn (0 days) female mice, unlike other flaviviruses [45].

## 8. Diagnosis

The laboratory diagnosis of suspected cases of OHFV infection can be performed after a differential diagnosis among other zoonotic infections (i.e., brucellosis or leptospirosis). Different diagnostic approaches are available, including serological and molecular tests. In the first phase of illness, characterized by aspecific mild symptoms, OHFV can be isolated from the blood of infected patients. In the second phase, where high fever and encephalitic symptoms emerge, the identification of the viral genome is more challenging, although the antibody titers are high [46]. Serological tests include the ELISA assay to search for antibodies against OHFV in serum samples and seroconversion tests conducted with neutralization, complement fixation, and hemagglutinin inhibition assays [47,48]. In a recent paper, it was demonstrated that OHFV NS1 recombinant protein could be used for the differential diagnosis of OHFV from TBE in an ELISA test due to their different antigenic properties, and it was demonstrated that the specificity of this ELISA test was about 90% [49]. ELISA and hemagglutination tests are the most sensitive methods during the first weeks of illness. The neutralization test could generate false-positive results due to its ability to show cross-neutralization between antibodies against several tick-borne flaviviruses and OHFV antibodies [32,50,51,52].

At a molecular level, Zhang et al. [53] have developed a specific SYBR Green-based RT-PCR assay specifically for the detection of OHFV infection. Previous assays were not designed specifically for OHFV and, in fact, could detect more flaviviruses [54], necessitating viral isolation or sequencing to verify the infection. This new, specific protocol is focused on the use of four primer pairs to detect all the different strains of OHFV. These primers anneal in the region of NS4A and NS4B of the OHFV genome, which is highly conserved in all OHFV strains but shows a good range of variability in the TBEV genome group [53]. This newly developed test shows a limit of detection of 10 copies of the OHFV genome and a minimum of 0.005 ng of total cDNA. Also, the specificity of this diagnostic protocol is high, showing a cycle threshold of 13.76 for OHFV cDNA and a cycle threshold of 29 and 32 for other TBEV genomes.

## 9. Prevention

Due to its transmissibility and causing severe illness, OHFV is classified as a Biosafety Level 4 agent in several countries. Up to date, two cases of laboratory infections have been reported due to needle puncture or a contaminated aerosol [42], but the principal sources of exposure remain tick or mosquito bites and contact with animal fluids.

To prevent and reduce the risk of OHFV infection, common prevention measures include wearing DPIs for laboratory workers, gloves for hunters, and masks for agricultural workers in endemic areas, and wearing appropriate dress for the general population or nylon clothes for people that live in focal sites. The Expert Committee of the World Health Organization [27] indicates that repellent sprays help with bite prevention; above all, repellents with N, N-diethylmetatoluamide for skin use or solution with permethrin should be applied to clothes. An extensive disinfestation of the entire territory could also help greatly in reducing the spread of mosquitoes and ticks and, consequently, the circulation of the virus. In addition, people should avoid drinking goat milk that has not gone through a pasteurization process.

## 10. Vaccination

In 1948, Chumakov produced a formalin-inactivated vaccine based on brain tissue from experimentally infected mice. In both animal and human studies, this vaccine provided effective protection against OHFV; however, production was discontinued due to adverse reactions to the mouse brain components of the vaccines.

Due to their antigenic similarities, vaccines for TBE virus were used to prevent OHFV infection during the 1991 outbreak [53]. However, its use was approved by a special directive from the local government. The efficacy of this strategy was experimentally tested by Chidumayo and colleagues in 2013 [55] in both mice and humans vaccinated with the European tick-borne encephalitis vaccine [56]. This study demonstrated that all vaccinated mice showed full protection from OHFV infection. In addition, they observed that humans who received the complete dosage of the vaccine showed a seroconversion rate of about 86%.

To date, neither approved vaccine preparations nor clinical trials have been available to test vaccines for humans against OHFV. A very recent study by Alnuqaydan and Eisa [57] identified and proposed a good candidate viral epitope for vaccine production. The study was conducted entirely in silico. Using immunoinformatics tools for the design, simulation, and prediction of biochemical properties, molecular toxicity, and antigenicity, they were able to identify appropriate OHFV polyprotein epitopes for the development of an effective, stable, and safe vaccine. This multi-epitope peptide, linked with human beta defensine-2 molecules to increase antigenic and immunogenic efficacy, shows a high affinity for the host immunoreceptor in silico. It should remain bound at the TLR4 docked side, providing epitopes to the host immune response and assuring the activation of innate and long-term adaptive responses. This study highlights the importance of reverse vaccinology in identifying vaccine targets [57]. The success of this new vaccine design strategy has been demonstrated with the MenB vaccine against *Meningococcus B* and other vaccines against bacterial infections like *Streptococcus* and Methicillin-Resistant *Staphylococcus Aureus* (MRSA). The study identified promising CTL, HTL, and B cell epitopes for an OHFV vaccine using this method. This should accelerate vaccine development by allowing researchers to test these epitopes in animal models.

## 11. Treatments

Currently, there is no specific treatment for OHFV, and, therefore, supportive therapy is essential, such as rehydration, symptomatic therapy to relieve pain and reduce temperature, and monitoring vital signs and laboratory data. A balanced diet, the administration of potassium chloride, glucose, and vitamins K and C, and a prolonged period of convalescence time are all indicated [32].

Pharmacological treatment includes the use of antiviral drugs such as ribavirin and paracetamol to reduce fever and pain, rehydration medications (solutions such as Ringer), and glucocorticoids in the case of massive hemorrhagic syndrome.

Ribavirin was tested against OHFV in cell lines and animal models. High concentrations of the drug moderately inhibited viral replication in cell culture (up to 99.8% viral inhibition at the concentration of 250 μg/mL), while a single intramuscular administration in a dose of 200 μg showed moderate efficacy against an experimental virus model (the survival rate of white mice was 55%). The disease is usually mild in chinchilla rabbits, and treatment with ribavirin reduces the duration of the disease by 2 days. Ribavirin toxicity was evaluated in chinchilla rabbits and common white mice weighing 6–8 g, and the maximum tolerated doses were 700 and 171 mg/kg, respectively [58].

The efficacy of several drugs against OHFV has been tested both in vitro and in vivo. Screening studies revealed that high concentrations of drugs able to induce interferons, like Larifan (bacteriophage-derived double-stranded RNA), could slow down and decrease viral replication. It has also been observed that human recombinant interferon α-2b can completely inhibit viral replication in cell culture conditions. No human studies are in progress, but in animal models, it is well documented that Larifan shows higher antiviral efficacy [59]. The strong antiviral activity of Larifan was demonstrated against the *Ondatra* strain of OHFV in animal studies. It protected 65% of infected mice from death and significantly reduced disease severity in rabbits. However, its antiviral effect was weak in cell cultures [60].

Though rare, OHFV can cause varying degrees of deafness, alopecia, and behavioral or psychological alterations in patients with neurological disease, and in some cases, long-term supportive care is needed (as reported by CDC guidelines [16]).

A more promising drug strategy to treat flaviviruses is represented by a class of synthetic peptides able to bind flavivirus envelop proteins, inhibiting two essential phases of the viral cycle: entry and virion assembly [61].

## 12. Future Perspectives and Open Questions

OHFV research is limited by the restricted endemic areas of the virus and the resulting scarcity of dedicated laboratories and data. Further investigations into the mechanisms of infection, immune evasion strategies, novel drug and treatment approaches, and vaccine development are crucial in advancing our understanding and the prevention of this disease. Several key questions remain unanswered. While the ED3 domain of the viral E protein is implicated in host cell binding and membrane fusion [62,63,64], the specific host cell receptor for OHFV and the dynamics of viral anti-receptor/receptor interactions remain unknown, representing a promising target for drug and antiviral molecules. Similarly, while the pH-based mechanism of E-prM heterodimer dissociation in the Golgi apparatus, crucial in virion formation and infectivity, has been described in other tick-borne encephalitis flaviviruses [65,66], a deeper understanding of this process in OHFV might reveal a possible target for therapeutic intervention. In a similarity study on the UniProt database, OHFV exhibited inefficient prM-E cleavage, resulting in virions with uncleaved prM. This uncleaved prM is hypothesized to contribute to immune evasion.

Furthermore, the potential impact of climate change on the geographic distribution of OHFV warrants investigation. Changes in bird migration patterns and mammal habits could expand the spread of infected vectors, increasing the population at risk. Therefore, studying the interplay between climate change and OHFV transmission is essential in proactive prevention strategies. A more comprehensive understanding of host–pathogen interactions, the precise mechanisms of infection and replication, the strategies employed for immune evasion, and the development of effective vaccines are all crucial in mitigating the threat posed by OHFV.

## 13. Conclusions

OHFV is a poorly investigated virus detected in a restricted endemic area. There may be new outbreaks of OHFV in the future, and it is necessary to undertake genomic and proteomic studies to open up new avenues in the prevention of infection, management, and therapy in this viral disease.

## Figures and Tables

**Table 1:** OHFV complete genome sequence information. In the table, for each complete genome sequence, GenBank accession number, source location, sequencing data, year and source of isolation, as well as the strain and genotype, are reported. The rows highlighted in orange are those related to strains isolated from human blood. Genotypes marked with an * indicate a classification assigned according to our tree but not previously reported in the literature [13].

GenBank Accession	Source Location	Sequencing Date	Isolation Year	Source of Isolation	Strain	Genotype [13]
AB507800	Unknown, Russia	20/07/2022	1948?	Human blood	Guriev	2
AY438626.1	Sargatski district, Omsk Region, Russia	15/10/2003	1947	Human blood	Kubrin	1
MT354615.1	Ust’-Tarka, Novosibirsk Region, Russia	16/04/2020	1990	*O. zibethicus*	P-15/2213	1 *
MT354616.1	Nazyvaevsk locality, Omsk Region, Russia	16/04/2020	2004	*O. zibethicus*	OZ-97/11285	3
MT354617.1	Nazyvaevsk locality, Omsk Region, Russia	16/04/2020	2004	*O. zibethicus*	OZ-96/11293	3
MT354618.1	Vengerovo Locality, Novobrisk Region, Russia	16/04/2020	1991	*O. zibethicus*	M-19/5099	1
MT354619.1	Krutinka Locality, Omsk Region, Russia	16/04/2020	1962	*D. marginatus*	Kabyrdak-39/10944	1
MT354620.1	Vengerovo Locality, Novobrisk Region, Russia	16/04/2020	1991	Mosquitoes	G17/10783	3
MT354621.1	Krasnozerskoe Locality, Novobrisk Region, Russia	16/04/2020	1963	*O. zibethicus*	Veselovka-753/11084	1
MT354622.1	Krutinka Locality, Omsk Region, Russia	16/04/2020	2000	*O. zibethicus*	B-41/9687	1
MT354623.1	Krutinka Locality, Omsk Region, Russia	16/04/2020	1999	*O. zibethicus*	B-37/9866	1
MT354624.1	Chany Locality, Novobrisk Region, Russia	16/04/2020	1990	*O. zibethicus*	B-30/10146	1
MT354625.1	Shuchje Lake, Kurgan Region, Russia	16/04/2020	1992	*O. zibethicus*	B-1/10186	3
MT354626.1	Vengerovo Locality, Novobrisk Region, Russia	16/04/2020	1991	*M. oeconomus*	pr.1007/10817	1
MT354627.1	Krutinka Locality, Omsk Region, Russia	16/04/2020	2007	*O. zibethicus*	362BI/362BI	1
MT354628.1	Krutinka Locality, Omsk Region, Russia	16/04/2020	1999	*O. zibethicus*	42M/9722	1
MT354629.1	Krutinka Locality, Omsk Region, Russia	16/04/2020	2002	*O. zibethicus*	17N/11153	1
MW773725.1	Nazyvaevsk locality, Omsk Region, Russia	18/03/2021	2004	*O. zibethicus*	OZ-97A	3 *
MW847419.1	Unknown, Russia	31/03/2021	1960	*O. zibethicus*	Nikitina	1 *
NC_005062.1	Unknown, Russia	09/12/2002	1947	Human blood	Bogoluvovska	1 *
OL689364.1	Tenis Lake, Omsk Region, Russia	30/11/2021	2007	*O. zibethicus*	Liv-356-0/13967	1 *
OL689365.1	Tenis Lake, Omsk Region, Russia	30/11/2021	2007	*O. zibethicus*	Liv-356-1/13955	1 *
OL689366.1	Tenis Lake, Omsk Region, Russia	30/11/2021	2007	*O. zibethicus*	Br-356-1/13959	1 *
OL689367.1	Tenis Lake, Omsk Region, Russia	30/11/2021	2007	*O. zibethicus*	Br-356-2/13971	1 *
OL689368.1	Tenis Lake, Omsk Region, Russia	30/11/2021	2007	*O. zibethicus*	Br-356-0/13957	1 *
OL689369.1	Tenis Lake, Omsk Region, Russia	30/11/2021	2007	*O. zibethicus*	Kid-362/13944	1 *
OL689370.1	Tenis Lake, Omsk Region, Russia	30/11/2021	2007	*O. zibethicus*	Liv-362/13943	1 *
OL689371.1	Tenis Lake, Omsk Region, Russia	30/11/2021	2007	*O. zibethicus*	Liv-362/13952	1 *
OL689372.1	Tenis Lake, Omsk Region, Russia	30/11/2021	2007	*O. zibethicus*	Bl-352/13981	1 *
OL689373.1	Tenis Lake, Omsk Region, Russia	30/11/2021	2007	*O. zibethicus*	Br-352/11656	1 *
OL689374.1	Tenis Lake, Omsk Region, Russia	30/11/2021	2007	*O. zibethicus*	Ur-356-0/13977	1 *
OL689375.1	Tenis Lake, Omsk Region, Russia	30/11/2021	2007	*O. zibethicus*	Ur-356-1/13980	1 *
OL689376.1	Tenis Lake, Omsk Region, Russia	30/11/2021	2007	*O. zibethicus*	Br-356-1/13969	1 *
OL689377.1	Tenis Lake, Omsk Region, Russia	30/11/2021	2007	*O. zibethicus*	Br-356-2/13964	1 *
OL689378.1	Tenis Lake, Omsk Region, Russia	30/11/2021	2007	*O. zibethicus*	Ur-353/13965	1 *
OL689379.1	Tenis Lake, Omsk Region, Russia	30/11/2021	2007	*O. zibethicus*	Br-353-0/11661	1 *
OL689380.1	Tenis Lake, Omsk Region, Russia	30/11/2021	2007	*O. zibethicus*	Br-353-1/13973	1 *
OL689381.1	Tenis Lake, Omsk Region, Russia	30/11/2021	2007	*O. zibethicus*	Bl-351/11704	1 *
OL689382.1	Tenis Lake, Omsk Region, Russia	30/11/2021	2007	*O. zibethicus*	Ur-353/13977	1
OL689383.1	Tenis Lake, Omsk Region, Russia	30/11/2021	2007	*O. zibethicus*	Br-352/352	1 *
OL689384.1	Tenis Lake, Omsk Region, Russia	30/11/2021	2007	*O. zibethicus*	Br-361/361	1 *
OL689385.1	Tenis Lake, Omsk Region, Russia	30/11/2021	2007	*O. zibethicus*	Br-361/13948	1 *
OL689386.1	Tenis Lake, Omsk Region, Russia	30/11/2021	2007	*O. zibethicus*	Ur-359/13965	1 *
OL689387.1	Tenis Lake, Omsk Region, Russia	30/11/2021	2007	*O. zibethicus*	Br-354/354	1 *
OL689388.1	Tenis Lake, Omsk Region, Russia	30/11/2021	2007	*O. zibethicus*	Br-354/11661	1 *
OP292291.1	Nazyvaevsk locality, Omsk Region, Russia	23/08/2022	2004	*O. zibethicus*	MO-1007	1 *
AB507800	Unknown, Russia	20/07/2022	1948?	Human blood	Guriev	2
AY438626.1	Sargatski district, Omsk Region, Russia	15/10/2003	1947	Human blood	Kubrin	1
MT354615.1	Ust’-Tarka, Novosibirsk Region, Russia	16/04/2020	1990	*O. zibethicus*	P-15/2213	1 *
